# Psychometric performance of the OECD Patient-Reported Indicator Surveys Patient Questionnaire (PaRIS-PQ) among adults with chronic conditions in Norway

**DOI:** 10.1007/s11136-026-04429-z

**Published:** 2026-11-07

**Authors:** Hilde Hestad Iversen, Oyvind Bjertnaes, Lina Harvold Ellingsen-Dalskau, Ian Porter, Candan Kendir, Jose M. Valderas

**Affiliations:** 1 Norwegian Institute of Public Health, PO Box 222, Skøyen, Oslo 0213 Norway; 2 Exeter Collaboration for Academic Primary Care (APEx), University of Exeter Medical School, St Luke’s Campus, Heavitree Road, Exeter, EX1 2LU UK; 3 Organisation for Economic Co-operation and Development (OECD), 2 rue André Pascal, Paris, 75016 France; 4 Amsterdam UMC, Meibergdreef 9, Amsterdam, 1105 AZ the Netherlands; 5 National University of Singapore, 21 Lower Kent Ridge Road, Singapore, 119077 Singapore; 6 National University Health System, 1E Kent Ridge Road, Singapore, 119228 Singapore

**Keywords:** Psychometrics, Primary Health Care, Chronic Disease, Patient-reported experience measures, Patient-reported outcome measures, Surveys and Questionnaires

## Abstract

**Purpose:**

The OECD Patient-Reported Indicator Surveys (PaRIS) initiative aims to provide standardised, internationally comparable patient-reported indicators for people living with chronic conditions receiving primary or generalist ambulatory care. This study evaluated the psychometric performance of the multi-item scales in the Norwegian PaRIS Patient Questionnaire (PaRIS-PQ).

**Methods:**

Data were drawn from the 2023 Norwegian PaRIS Main Survey. Respondents aged ≥ 45 years with at least one self-reported chronic condition were included. Measures were PROMIS Global Physical Health, PROMIS Global Mental Health, and WHO-5 Well-Being Index as patient-reported outcome measures (PROMs); the Person-Centred Coordinated Care Experience Questionnaire (P3CEQ) as a patient-reported experience measure (PREM); and the Porter–Novelli Scale (PNS) and self-management confidence as capability-related measures. Analyses assessed structural validity, internal consistency reliability, measurement invariance, item functioning, and construct validity.

**Results:**

Of 8,683 respondents, 6,691 reported at least one chronic condition. PROM scales performed best, with acceptable internal consistency (α = 0.80–0.89) and strongest confirmatory support for PROMIS Global Physical Health (CFI = 0.992, RMSEA = 0.080). The PREM (P3CEQ) showed weaker performance, with suboptimal fit for the total scale (CFI = 0.818, RMSEA = 0.132), limited scalar invariance, and low internal consistency for the care coordination subscale (α = 0.61). Capability-related measures showed mixed performance. Metric invariance was supported across all tested models, whereas scalar invariance was less consistent. Construct validity findings were consistent with expectations.

**Conclusion:**

PROM scales showed the most consistent psychometric support. PREM and capability-related measures were more heterogeneous and require cautious, scale-specific interpretation, especially in subgroup comparisons and when used as latent measures. Evaluation and refinement of weaker experience- and capability-related measures is warranted.

**Supplementary Information:**

The online version contains supplementary material available at https://doi.org/10.1007/s11136-026-04429-z.

## Background

Health systems worldwide face increasing pressures from ageing populations, rising multimorbidity and the growing number of people living with chronic conditions [1]. Chronic conditions substantially affect functioning, quality of life, and account for a major share of health-care utilisation and expenditures across countries [2]. Many countries are therefore redesigning health systems towards more people-centred and integrated care, with primary care playing a pivotal role in prevention, long-term management, coordination, continuity and self-management support [3]. Despite this role, comprehensive and comparable information on how people with chronic conditions experience their care, and how well services support their functioning, well-being and ability to manage their health, remains limited [4].

Patient-reported outcome measures (PROMs) and patient-reported experience measures (PREMs) complement clinical and administrative data by capturing people’s functioning, well-being, capabilities and experiences of care [5]. However, many existing measures focus on single diseases, specific interventions or isolated encounters and may fail to capture central elements of person-centred and coordinated care for individuals receiving services across multiple providers [2, 4, 6]. This limits the coverage and comparability of patient-reported indicators within and across health systems.

To address these gaps, the Organisation for Economic Co-operation and Development (OECD) launched the Patient-Reported Indicator Surveys (PaRIS), which aim to strengthen international healthcare performance measurement by focusing on outcomes and care experiences among people living with chronic conditions [2]. PaRIS comprises a patient questionnaire and a primary care practice questionnaire designed to support cross-country comparison of patient-reported indicators among adults aged 45 years and older receiving primary or generalist ambulatory care in light of practice characteristics [6–8]. Patient-level information from the PaRIS Patient Questionnaire (PaRIS-PQ) [9] is complemented by organisational and provider-reported data from the PaRIS Primary Care Practice Questionnaire (PaRIS-PCPQ) [8]. The PaRIS conceptual framework links patient-reported outcomes and experiences to characteristics of primary care delivery, health system design, policy and context, enabling analyses of how practice- and system-level factors are associated with patients’ outcomes and experiences across countries and population subgroups [6].

The PaRIS-PQ integrates established scales with targeted items covering remaining subdomains of the PaRIS framework [9]. The OECD flagship report summarises core domains through the “PaRIS-10” key indicators: physical, mental and social functioning, well-being, general health, confidence to self-manage, experienced coordination, person-centred care, experienced quality and trust [2]. The usefulness of these indicators depends on the psychometric robustness of the underlying multi-item scales from which several are constructed.

International development and Field Trial evaluation of the PaRIS-PQ have provided initial support for the questionnaire, including acceptable patient-level reliability for most measures [9, 10]. However, more restrictive forms of cross-country measurement invariance were not fully supported, and the Field Trial findings informed revisions to the questionnaire before implementation of the Main Survey [9, 10]. The Field Trial therefore provided an important foundation but did not establish the measurement properties of the final questionnaire within each national survey population.

Norway participated in both the Field Trial and the 2023 Main Survey. In Norway, the national general practitioner (GP) registry was used to establish a provider-linked, registry-informed sampling design among people with chronic conditions. National findings were comparatively favourable for patients’ well-being and trust in the health system, but weaker for confidence in managing their own health and experienced coordination [11]. Previous analyses of Norwegian data also showed potential selection effects related to GP participation [12].

Even when measures are developed within an international survey programme, psychometric evaluation in new national and linguistic contexts remains important because measures translated or transferred across settings may not retain equivalent dimensionality, reliability, or measurement invariance [13]. The measurement properties of the multi-item scales in the Norwegian PaRIS-PQ have not yet been evaluated using Main Survey data. Such evaluation is therefore important to support interpretation of Norwegian PaRIS results and comparisons across population subgroups.

Based on the Norwegian PaRIS Main Survey data, the aim of this study was to evaluate the psychometric performance of the multi-item scales included in the Norwegian PaRIS-PQ among persons aged 45 years and older with one or more chronic conditions. Psychometric evaluation included structural validity, internal consistency reliability, construct validity, measurement invariance across key sociodemographic groups, and item-level performance. The findings may inform scale-specific interpretation of Norwegian PaRIS results, subgroup comparisons and further refinement of PaRIS measures.

## Methods

### Data collection and sample

The present study was a psychometric evaluation using cross-sectional data from the 2023 Norwegian PaRIS Main Survey, conducted as part of the OECD PaRIS [2, 9]. PaRIS is designed to assess health outcomes and care experiences of primary care users, with a focus on people with chronic conditions. PaRIS includes respondents aged 45 years and older who had at least one contact with their primary care practice in the past six months. Given its design feature, the present psychometric analyses were restricted to respondents reporting at least one chronic condition.

Data collection followed the PaRIS study protocol [7]. In Norway, the national GP Registry, which covers all regular GPs and almost the entire population, was used for the first stage of the provider-linked sampling procedure [11, 14]. A random sample of 250 GPs was invited to participate in PaRIS and complete the PaRIS-PCPQ. Of these, 121 responded; three were excluded because they had no or only one eligible patient or had stopped practising, leaving 118 participating GPs. GP questionnaire data were not included in the present psychometric analyses; GP participation served to define the sampling frame for the patient sample. In the second stage, eligible patients were identified using the Norwegian Control and Payment of Health Reimbursements Database (KUHR) [15] as those listed with a participating GP, aged 45 years or older, and with at least one consultation during the six months preceding sample selection.

Patient data collection took place in May–June 2023. Included patients (*n* = 19,637) were invited using a secure survey link and unique access code, primarily through Helsenorge [16]. Up to three SMS reminders were sent to digital non-respondents. Patients not registered for digital communication (12.3%) received a postal invitation and one postal reminder, both including a Norwegian paper questionnaire. Additional registry data from KUHR, the GP Register and Statistics Norway were linked to the survey data.

### Questionnaire and multi-item scales

The development of the PaRIS-PQ has been described elsewhere [9]. In brief, candidate measures were mapped to the PaRIS conceptual framework, appraised using the Evaluating the Measurement of Patient-Reported Outcomes (EMPRO) tool [17], refined through a modified Delphi process, and selected to ensure conceptual coverage while limiting respondent burden [6, 7, 9].

Existing Norwegian translations were incorporated for the Patient-Reported Outcomes Measurement Information System (PROMIS) Global Health item set, the WHO-5 Well-Being Index (WHO-5), and several original items from the Person-Centred Coordinated Care Experience Questionnaire (P3CEQ). The remaining content evaluated in the present study was translated or adapted through the centralised PaRIS translation process, using the Translation, Review, Adjudication, Pretesting and Documentation (TRAPD) approach to support conceptual equivalence across countries while allowing adaptation to the national context [18]. The assembled Norwegian PaRIS-PQ was cognitively tested in 20 interviews prior to field implementation. Because of the questionnaire length, cognitive testing used three separate interview protocols and did not assess the full patient questionnaire within a single interview. In the Main Survey, the PaRIS-PQ was available in the two official written standards of Norwegian, as well as English [11].

The present psychometric evaluation included the multi-item scales in the Norwegian PaRIS-PQ, summarised in Supplementary Table S1. The full 10-item PROMIS Global Health item set was administered. It comprises two four-item summary scales, PROMIS Global Physical Health (PROMIS GPH) and PROMIS Global Mental Health (PROMIS GMH), originally scored by averaging the four constituent items within each scale, while the two remaining items, general health and satisfaction with social roles, are recommended for separate reporting [19]. As the present study evaluated multi-item scales, only PROMIS GPH and PROMIS GMH were included in the psychometric analyses. The WHO-5 [20], the PaRIS version of the P3CEQ [21], and the 10-item Porter–Novelli Scale (PNS) [22] were administered in their entirety. For confidence in self-management, PaRIS included four items derived from the Medicare Current Beneficiary Survey (MCBS) [23] and one item from the eHealth Literacy Scale (eHEALS) [24]; the full eHEALS was not administered. Although originating from different measures, these items were evaluated together in the PaRIS Field Trial as a measure of self-management confidence within the broader health and healthcare capabilities domain [9, 10]. The MCBS items assess confidence in performing specific self-management tasks, whereas the eHEALS item assesses confidence in using online health information for health-related decision-making [23, 24]. Given their different source measures and content, the five-item MCBS + eHEALS specification was evaluated exploratorily alongside a four-item MCBS-only specification.

Single-item PaRIS measures were not designed as psychometric scales and were therefore not evaluated. The PROMIS GPH, PROMIS GMH and WHO-5 were treated as PROMs; the P3CEQ as a PREM; and the PNS and self-management confidence item set as measures within the health and healthcare capabilities domain [6, 9, 10].

### Statistical analysis

In line with the OECD PaRIS approach [9], the psychometric analyses evaluated reflective measurement specifications for the multi-item scales, item sets and empirically identified factors. Reflective models were prespecified for PROMIS GPH, PROMIS GMH and WHO-5, and for the proposed P3CEQ subscales [19–21]. The P3CEQ total scale was examined as a candidate reflective specification based on PaRIS Field Trial findings, whereas PNS factors and both self-management confidence specifications were evaluated exploratorily following empirical examination of dimensionality [9, 10]. In line with current COSMIN guidance, factor-analytic and item response theory (IRT) findings were interpreted as evidence of structural validity, while internal consistency and measurement invariance were interpreted within the same reflective measurement framework [25].

Structural validity was evaluated using confirmatory factor analysis (CFA) for scales with well-established hypothesised structures, and exploratory factor analysis (EFA) followed by CFA for scales with less certain dimensionality. EFA and CFA were conducted using the same dataset, without splitting the sample into separate exploratory and confirmatory subsets. PROMIS GPH and PROMIS GMH were evaluated primarily using CFA, consistent with the original validation study [19], and WHO-5 was likewise evaluated as a unidimensional scale [20]. For these prespecified PROM scales, EFA was used only to descriptively check whether item correlations supported the expected structures, whereas for P3CEQ, PNS and the self-management confidence item sets, EFA was used to examine the number and interpretation of factors before CFA models were specified.

The P3CEQ was originally conceptualised as comprising two related subdomains, Person-Centredness (P3CEQ-PC) and Care Coordination (P3CEQ-CC) [21]. However, PaRIS Field Trial analyses indicated high intercorrelations between the subdomains and a single dominant factor [9]. Therefore, both the total scale and proposed subscales were examined. For the PNS, Field Trial analyses identified two dimensions, active engagement and reliance on health professionals [9, 10]. EFA preceded confirmatory testing in the Norwegian sample. For self-management confidence, PaRIS Field Trial analyses operationalised a broader construct combining P3CEQ10, MCBS items and eHEALS [9]; here, P3CEQ10 was retained within the P3CEQ analyses, while the MCBS + eHEALS and MCBS-only specifications were examined separately.

EFA used principal axis factoring on Pearson correlation matrices with pairwise complete observations. Factorability was assessed using the Kaiser–Meyer–Olkin (KMO) statistic, with values ≥ 0.70 considered desirable, and Bartlett’s test of sphericity was required to be statistically significant [26–28]. Factor retention was guided by eigenvalues, scree plots and parallel analysis [28]. Items with primary loadings below 0.40 or cross-loadings above 0.30 were interpreted as showing limited factorial specificity [29]. Internal consistency reliability was assessed using Cronbach’s alpha (α), with values ≥ 0.70 considered acceptable [30].

CFA was used to test the prespecified or EFA-informed measurement models. Item responses were specified as ordered categorical and models were estimated using weighted least squares with mean and variance adjustment (WLSMV). Model fit was evaluated using the mean- and variance-adjusted χ² statistic, Comparative Fit Index (CFI), Tucker–Lewis Index (TLI), Root Mean Square Error of Approximation (RMSEA), and Standardised Root Mean Square Residual (SRMR). Because χ² is sensitive to large samples, model fit was assessed primarily using approximate fit indices. CFI and TLI ≥ 0.95, RMSEA < 0.07, and SRMR < 0.05 were used as reference criteria of good fit [31, 32]. For models with few degrees of freedom, RMSEA may be overly sensitive; fit indices were therefore interpreted jointly and considered alongside standardised factor loadings [33, 34].

Measurement invariance was evaluated for the multi-item scales and subscales retained for subgroup comparisons after the initial structural validity assessment. Multigroup CFA was conducted across gender, age group and educational level using the same WLSMV specification. For the ordered-categorical indicators, invariance was tested sequentially using configural, threshold, metric and scalar models, with increasingly restrictive equality constraints across groups. Model fit was assessed using the fit indices and criteria described above. Invariance was considered supported when decreases in CFI were ≤ 0.010 and increases in RMSEA were ≤ 0.015 relative to the less constrained model [35–37]. Metric invariance was interpreted as supporting comparisons of associations across groups, whereas scalar invariance was considered important for comparing latent means and interpreting observed subgroup differences.

Item-level performance was examined using IRT. Factor-analytic and IRT approaches were used to evaluate complementary aspects of psychometric performance: factor analyses assessed dimensionality and structural validity, while IRT assessed item-level functioning and measurement precision. For scales with EFA/CFA evidence of a dominant underlying dimension, graded response models (GRM) for ordered categorical items were estimated using marginal maximum likelihood; where structural support was mixed, IRT was applied exploratorily [38]. The GRM was selected because it accommodates ordered categorical responses and permits discrimination to vary across items. Discrimination (a), thresholds (b), global fit using the limited-information C2 statistic with RMSEA, Standardised Root Mean Square Residual (SRMSR) and CFI, and item fit using S-X² and item-level RMSEA were assessed [39–41]. Discrimination parameters were interpreted descriptively as very low (0.01–0.34), low (0.35–0.64), moderate (0.65–1.34), high (1.35–1.69), or very high (≥ 1.70); these categories were treated as approximate guidelines rather than strict quality criteria [42]. Local item dependence was examined using Yen’s Q3 residual correlations. Adjusted Q3 values were calculated by subtracting the mean off-diagonal Q3 from each item-pair residual correlation [43, 44]. Adjusted Q3 values above 0.20, based on unrounded estimates, were used as a heuristic screening value for possible local item dependence rather than as an absolute statistical criterion; detailed results are provided in Supplementary Table S2. Test information curves, item information curves, and category probability curves were inspected to evaluate measurement precision, information distribution, latent trait coverage, and response category functioning. An overview of the test information curves for all IRT models is provided in Supplementary Figure S1, and model-specific test information, item information, and category probability curves are provided in Supplementary Figures S2–S11.

Construct validity was assessed by testing a priori hypotheses regarding the magnitude of correlations among theoretically related and distinct scale scores, consistent with COSMIN guidance [25], the PaRIS conceptual framework and previous Field Trial analyses [6, 9, 10]. Pearson correlations were calculated between theoretically related outcome, experience, and capability-related domains, with Spearman correlations used as sensitivity analyses. PROMs were expected to correlate moderately to strongly with each other. PROM–PREM correlations were expected to be weak to moderate, PROM–capability correlations to be weak, and PREM–capability correlations to be weak to moderate.

All analyses were conducted in R version 4.2.3, using psych for EFA and internal consistency analyses, lavaan and semTools for CFA and measurement invariance testing, and mirt for IRT analyses.

## Results

### Sample

Of 8,683 respondents (response rate 44.2%), 8,536 had valid data on chronic conditions; of these, 6,691 (78.4%) reported at least one chronic condition and were included in the analyses (Table 1).

**Table 1:** Respondent characteristics of the analytical sample (≥1 chronic condition)

	*n*	%
**Gender (** ***n*** **= 6,691)**		
Women	3,667	54.8
Men	3,024	45.2
**Age group (years) (** ***n*** **= 6,691)**		
45–54	1,280	19.1
55–69	2,929	43.8
70–79	1,685	25.2
≥80	797	11.9
**Number of chronic conditions (** ***n*** **= 6,691)**		
1	2,969	44.4
2	2,088	31.2
≥3	1,634	24.4
**Education level (** ***n*** = 6,653)*		
Low	1,079	16.2
Medium	3,133	47.1
High	2,441	36.7

* Education level was classified according to Statistics Norway: low = primary and lower secondary education, including no completed education; medium = upper ​secondary and post-secondary vocational education; high = university or college education

### Factor analysis and internal consistency

EFA and reliability results for the P3CEQ, PNS and self-management confidence specifications are shown in Tables 2, 3, 4 and CFA results for all scales are shown in Table 5. For PROMIS GPH, PROMIS GMH and WHO-5, descriptive EFAs were compatible with the expected structures, and internal consistency was acceptable (α = 0.80–0.89) (results not shown). CFA results were strongest for PROMIS GPH, with CFI, TLI and SRMR meeting the reference criteria, while RMSEA was slightly above the criterion. PROMIS GMH and WHO-5 showed broadly supportive but mixed fit, with CFI and SRMR meeting the criteria but RMSEA and TLI not. Standardised factor loadings were moderate to strong across the three PROM models (0.66–0.90).

**Table 2:** Exploratory factor analysis and reliability statistics for the P3CEQ total scale, P3CEQ-PC subscale, and P3CEQ-CC subscale

Person-Centred Coordinated Care Experience Questionnaire	P3CEQ total scale Cronbach’s α = 0.728			P3CEQ-PC subscale Cronbach’s α = 0.745			P3CEQ-CC subscale Cronbach’s α = 0.606		
	Factor loading	Corrected item–total correlation	Cronbach’s α if item deleted	Factor loading	Corrected item–total correlation	Cronbach’s α if item deleted	Factor loading	Corrected item–total correlation	Cronbach’s α if item deleted
P3CEQ 1 Discussion of what matters most	0.550	0.488	0.695	0.515	0.445	0.718			
P3CEQ 2 Involvement in care decisions	0.655	0.504	0.698	0.675	0.554	0.704			
P3CEQ 3 Being treated as a whole person	0.659	0.505	0.698	0.683	0.552	0.704			
P3CEQ 4 Having to repeat information	0.441	0.320	0.716	0.468	0.375	0.730			
P3CEQ 5 Care organised to meet patient needs	0.714	0.571	0.690	0.719	0.588	0.698	0.548	0.407	0.560
P3CEQ 6 Single professional coordinating care	0.352	0.335	0.728				0.444	0.347	0.571
P3CEQ 7 Care plan addressing patient needs	0.331	0.330	0.724				0.449	0.361	0.555
P3CEQ 8 Support for self-management	0.520	0.442	0.697	0.516	0.466	0.718	0.530	0.352	0.557
P3CEQ 9 Timely and useful self-management information	0.481	0.455	0.694	0.439	0.395	0.734	0.577	0.432	0.517
P3CEQ 10 Confidence in managing own health	0.331	0.247	0.725	0.354	0.305	0.742			

*Abbreviations*: *EFA *exploratory factor analysis, *P3CEQ* Person-Centred Coordinated Care Experience Questionnaire, *P3CEQ-PC* P3CEQ Person-Centredness subscale, *P3CEQ-CC* P3CEQ Care Coordination subscale

Total analytic sample = 6,691. EFAs were based on pairwise complete observations; harmonic N was 6,198 for the P3CEQ total scale, 6,122 for the P3CEQ-PC subscale, and 6,436 for the P3CEQ-CC subscale

**Table 3:** Exploratory factor analysis and reliability statistics for the PNS

Porter-Novelli Scale	PNS Factor 1: Active engagement Cronbach’s α = 0.738			PN​S Factor 2: Health information comprehension Cronbach’s α = 0.795			PNS Factor 3: Reliance and cooperation with health professionals Cronbach’s α = 0.581		
	Factor loadings	Corrected item–total correlation	Cronbach’s α if item deleted	Factor loadings	Corrected item–total correlation	Cronbach’s α if item deleted	Factor loadings	Corrected item–total correlation	Cronbach’s α if item deleted
PNS 1 Reliance on professionals for health information							0.573	0.373	0.502
PNS 2 Perceived complexity of health issues				0.821	0.660	0.643			
PNS 3 Active disease prevention	0.473	0.438	0.733						
PNS 4 Reliance on professionals for health decisions							0.448	0.296	0.569
PNS 5 Importance of health information	0.725	0.565	0.657						
PNS 6 Health information for oneself and family	0.831	0.606	0.633						
PNS 7 Difficulty understanding written health information				0.829	0.660	0.678			
PNS 8 Working with professionals to manage health							0.681	0.486	0.417
PNS 9 Discussing relevant information with professionals							0.341	0.315	0.545
PNS 10 Understanding personal health risks	0.598	0.526	0.685						

*Abbreviations*: *EFA* exploratory factor analysis, *PNS* Porter–Novelli Scale

Total analytic sample = 6,691. EFA was based on pairwise complete observations; harmonic *N* = 6,634

**Table 4:** Exploratory factor analysis and reliability statistics for MCBS items combined with the eHEALS item and for the MCBS items alone

Patients’ confidence in self-management	MCBS + eHEALS Cronbach’s α = 0.772			MCBS Cronbach’s α = 0.788		
	Factor loadings	Corrected item–total correlation	Cronbach’s α if item deleted	Factor loadings	Corrected​item–total correlation	Cronbach’s α if item deleted
MCBS 1 Confidence in following home-care instructions	0.758	0.619	0.705	0.787	0.649	0.709
MCBS 2 Confidence in following lifestyle-change instructions	0.719	0.588	0.716	0.743	0.615	0.726
MCBS 3 Confidence in recognising need for medical care	0.704	0.612	0.707	0.684	0.608	0.729
MCBS 4 Confidence in recognising medication side effects	0.613	0.546	0.731	0.578	0.516	0.777
eHEALS Confidence in using online health information for decisions	0.407	0.369	0.788			

*Abbreviations*: *EFA* exploratory factor analysis, *MCBS* Medicare Current Beneficiary Survey, *eHEALS* eHealth Literacy Scale

Total analytic sample = 6,691. EFAs were based on pairwise complete observations; harmonic N was 5,801 for the MCBS + eHEALS specification and 6,264 for the MCBS-only specification

**Table 5:** Confirmatory factor analysis for all scales and subscales

Model	Chi-square (χ²)	df	*p*	CFI	TLI	RMSEA	SRMR	Standardised factor loadings
**PROMIS GPH (** ***n*** ** = 6688)**	57.69	2	< 0.001	0.992	0.977	0.080	0.015	
PROMIS GPH 1 Physical health								0.818
PROMIS GPH 2 Everyday physical functioning								0.823
PROMIS GPH 3 Average fatigue								0.752
PROMIS GPH 4 Average pain								0.663
**PROMIS GMH (** ***n*** ** = 6689)**	293.01	2	< 0.001	0.973	0.919	0.167	0.028	
PROMIS GMH 1 Quality of life								0.785
PROMIS GMH 2 Mental health								0.867
PROMIS GMH 3 Satisfaction with social activities and relationships								0.822
PROMIS GMH 4 Emotional problems								0.719
**WHO-5 (** ***n*** ** = 6686)**	829.95	5	< 0.001	0.960	0.920	0.167	0.030	
WHO-5 1 Feeling cheerful and in good spirits								0.903
WHO-5 2 Feeling calm and relaxed								0.863
WHO-5 3 Feeling active and vigorous								0.813
WHO-5 4 Interest in daily life								0.806
WHO-5 5 Feeling refreshed on waking								0.765
**P3CEQ total scale (** ***n*** ** = 6691)**	2015.57	35	< 0.001	0.818	0.766	0.132	0.078	
P3CEQ 1 Discussion of what matters most								0.623
P3CEQ 2 Involvement in care decisions								0.753
P3CEQ 3 Being treated as a whole person								0.781
P3CEQ 4 Having to repeat information								0.500
P3CEQ 5 Care organised to meet patient needs								0.807
P3CEQ 6 Single professional coordinating care								0.451
P3CEQ 7 Care plan addressing patient needs								0.458
P3CEQ 8 Support for self-management								0.572
P3CEQ 9 Timely and useful self-management information								0.555
P3CEQ 10 Confidence in managing own health								0.375
**P3CEQ-PC subscale (** ***n*** ** = 6690)**	1075.21	20	< 0.001	0.895	0.853	0.120	0.058	
P3CEQ 1 Discussion of what matters most								0.598
P3CEQ 2 Involvement in care decisions								0.768
P3CEQ 3 Being treated as a whole person								0.794
P3CEQ 4 Having to repeat information								0.524
P3CEQ 5 Care organised to meet patient needs								0.804
P3CEQ 8 Support for self-management								0.577
P3CEQ 9 Timely and useful self-management information								0.519
P3CEQ 10 Confidence in managing own health								0.400
**P3CEQ-CC subscale (** ***n*** ** = 6680)**	263.16	5	< 0.001	0.891	0.783	0.147	0.057	
P3CEQ 5 Care organised to meet patient needs								0.665
P3CEQ 6 Single professional coordinating care								0.540
P3CEQ 7 Care plan addressing patient needs								0.583
P3CEQ 8 Support for self-management								0.595
P3CEQ 9 Timely and useful self-management information								0.630
**PNS three-factor model (** ***n*** ** = 6684)**	4088.07	32	< 0.001	0.891	0.847	0.107	0.085	
PNS 1 Reliance on professionals for health information								0.466
PNS 2 Perceived complexity of health issues								0.863
PNS 3 Active disease prevention								0.590
PNS 4 Reliance on professionals for health decisions								0.207
PNS 5 Importance of health information								0.789
PNS 6 Health information for oneself and family								0.810
PNS 7 Difficulty understanding written health information								0.846
PNS 8 Working with professionals to manage health								0.839
PNS 9 Discussing relevant information with professionals								0.618
PNS 10 Understanding personal health risks								0.746
**MCBS + eHEALS (** ***n*** ** = 6680)**	1073.52	5	< 0.001	0.884	0.768	0.214	0.069	
MCBS 1 Confidence in following home-care instructions								0.868
MCBS 2 Confidence in following lifestyle-change instructions								0.823
MCBS 3 Confidence in recognising need for medical care								0.722
MCBS 4 Confidence in recognising medication side effects								0.652
eHEALS Confidence in using online health information for decisions								0.441
**MCBS (** ***n*** ** = 6678)**	839.64	2	< 0.001	0.904	0.713	0.290	0.067	
MCBS 1 Confidence in following home-care instructions								0.874
MCBS 2 Confidence in following lifestyle-change instructions								0.828
MCBS 3 Confidence in recognising need for medical care								0.716
MCBS 4 Confidence in recognising medication side effects								0.636

*Abbreviations*: χ² chi-square statistic, *df* degrees of freedom, *CFI* Comparative Fit Index, *TLI* Tucker–Lewis Index, *RMSEA* Root Mean Square Error of Approximation, *SRMR* Standardised Root Mean Square Residual, *PROMIS GPH* Patient-Reported Outcomes Measurement Information System Global Physical Health, *PROMIS GMH* Patient-Reported Outcomes Measurement Information System Global Mental Health, *WHO-5* WHO-5 Well-Being Index, *P3CEQ* Person-Centred Coordinated Care Experience Questionnaire, *P3CEQ-PC* P3CEQ Person-Centredness subscale, *P3CEQ-CC* P3CEQ Care Coordination subscale, *PNS* Porter–Novelli Scale, *MCBS* Medicare Current Beneficiary Survey, *eHEALS* eHealth Literacy Scale, *WLSMV* weighted least squares with mean and variance adjustment

Models were estimated using WLSMV with indicators treated as ordered categorical; the mean- and variance-adjusted χ² statistic and robust CFI, TLI and RMSEA are reported

For the P3CEQ total scale, P3CEQ-PC subscale and P3CEQ-CC subscale, EFA factorability was acceptable to good (KMO = 0.72–0.84; Bartlett’s tests all *p* < 0.001). Eigenvalues suggested two factors for the P3CEQ total scale, but the two-factor solution lacked conceptual clarity and showed substantial cross-loadings. Consistent with the PaRIS Field Trial [9], a one-factor solution was retained, explaining 27% of the variance (α = 0.73), with three items loading below 0.40. For the P3CEQ-PC subscale, evidence for a second factor was limited, and the subscale was treated as unidimensional (31% variance explained; α = 0.75). For the P3CEQ-CC subscale, EFA supported a one-factor solution, but internal consistency was below criterion (26% variance explained; α = 0.61). Corresponding CFA results showed suboptimal fit for the P3CEQ total scale and both subscales; none of the models met the prespecified global fit criteria.

The PNS items showed acceptable factorability (KMO = 0.75; Bartlett’s *p* < 0.001), and EFA supported a three-factor structure explaining 44% of the variance: active engagement, health information comprehension, and reliance and cooperation with health professionals. Internal consistency was acceptable for PNS Factors 1 and 2 (α = 0.74 and 0.80), but below criterion for PNS Factor 3 (α = 0.58). In CFA, the correlated three-factor model did not meet the prespecified global fit criteria, although standardised factor loadings suggested broadly interpretable dimensions.

The self-management confidence specifications showed acceptable EFA factorability (KMO = 0.76 and 0.73; Bartlett’s tests both *p* < 0.001), with one-factor solutions explaining 43% of the variance for MCBS + eHEALS and 49% for MCBS-only. Internal consistency was acceptable for both specifications (α = 0.77 and 0.79). In CFA, neither model provided clear confirmatory support for robust unidimensional measurement; MCBS item loadings were moderate to strong in both models, while the eHEALS item showed the weakest loading in the five-item model.

### Measurement invariance

Measurement invariance results are shown in Table 6. Threshold and metric invariance criteria were met across all tested models and grouping variables, although absolute model fit was weaker for several P3CEQ, PNS Factor 3 and self-management confidence models.

**Table 6:** Fit indices for measurement invariance models for all scales across gender, age group, and educational level

	Chi-square (χ²)	df	CFI	RMSEA	SRMR	ΔCFI	ΔRMSEA
**PROMIS GPH**							
**Gender (** ***n*** ** = 6688)**							
Configural	55.93	4	0.997	0.062	0.015	–	–
Threshold	68.67	12	0.997	0.038	0.015	0.000	−0.025
Metric	83.57	15	0.997	0.037	0.018	−0.001	−0.001
Scalar	129.22	18	0.995	0.043	0.018	−0.002	0.006
**Age groups (** ***n*** ** = 6688)**							
Configural	62.27	8	0.998	0.064	0.015	–	–
Threshold	100.89	32	0.997	0.036	0.015	−0.001	−0.028
Metric	138.74	41	0.996	0.038	0.018	−0.001	0.002
Scalar	475.71	50	0.981	0.071	0.023	−0.015	0.034
**Educational level (** ***n*** ** = 6650)**							
Configural	72.91	6	0.996	0.071	0.018	–	–
Threshold	90.49	22	0.996	0.037	0.018	0.000	−0.033
Metric	111.30	28	0.996	0.037	0.020	−0.001	−0.001
Scalar	140.75	34	0.994	0.038	0.021	−0.001	0.001
**PROMIS GMH**							
**Gender (** ***n*** ** = 6689)**							
Configural	303.22	4	0.991	0.150	0.029	–	–
Threshold	243.31	12	0.993	0.076	0.029	0.002	−0.074
Metric	236.24	15	0.993	0.066	0.030	0.000	−0.010
Scalar	353.59	18	0.990	0.075	0.029	−0.003	0.008
**Age groups (** ***n*** ** = 6689)**							
Configural	291.57	8	0.992	0.146	0.028	–	–
Threshold	284.39	32	0.992	0.069	0.028	0.001	−0.077
Metric	279.40	41	0.993	0.059	0.029	0.000	−0.010
Scalar	571.02	50	0.985	0.079	0.029	−0.008	0.020
**Educational level (** ***n*** ** = 6651)**							
Configural	295.91	6	0.991	0.148	0.029	–	–
Threshold	285.18	22	0.992	0.073	0.029	0.001	−0.074
Metric	270.25	28	0.993	0.062	0.030	0.001	−0.011
Scalar	405.21	34	0.989	0.070	0.030	−0.004	0.008
**WHO-5**							
**Gender (** ***n*** ** = 6686)**							
Configural	850.29	10	0.989	0.159	0.031	–	–
Threshold	635.21	25	0.992	0.085	0.031	0.003	−0.073
Metric	634.73	29	0.992	0.079	0.032	0.000	−0.006
Scalar	687.50	33	0.991	0.077	0.032	−0.001	−0.002
**Age groups (** ***n*** ** = 6686)**							
Configural	870.56	20	0.989	0.160	0.033	–	–
Threshold	751.08	62	0.991	0.082	0.033	0.002	−0.078
Metric	675.19	74	0.992	0.070	0.033	0.001	−0.012
Scalar	1085.45	86	0.987	0.083	0.033	−0.005	0.014
**Educational level (** ***n*** ** = 6648)**							
Configural	799.49	15	0.990	0.154	0.031	–	–
Threshold	635.41	45	0.992	0.077	0.031	0.003	−0.077
Metric	591.12	53	0.993	0.068	0.031	0.001	−0.009
Scalar	720.01	61	0.991	0.070	0.031	−0.002	0.002
**P3CEQ total scale**							
**Gender (** ***n*** ** = 6691)**							
Configural	2048.82	70	0.917	0.092	0.079	–	–
Threshold	2116.65	78	0.915	0.088	0.079	−0.002	−0.004
Metric	1710.90	87	0.932	0.075	0.079	0.017	−0.014
Scalar	1957.08	94	0.922	0.077	0.080	−0.010	0.002
**Age groups (** ***n*** ** = 6691)**							
Configural	2070.70	140	0.920	0.091	0.080	–	–
Threshold	2160.64	164	0.918	0.085	0.080	−0.003	−0.005
Metric	1778.84	191	0.935	0.071	0.081	0.017	−0.015
Scalar	2304.70	212	0.914	0.077	0.082	−0.021	0.006
**Educational level (** ***n*** ** = 6653)**							
Configural	2034.41	105	0.919	0.091	0.079	–	–
Threshold	2134.21	121	0.915	0.087	0.079	−0.004	−0.004
Metric	1660.61	139	0.936	0.070	0.079	0.021	−0.016
Scalar	2150.01	153	0.916	0.077	0.081	−0.020	0.006
**P3CEQ-PC subscale**							
**Gender (** ***n*** ** = 6690)**							
Configural	1112.22	40	0.948	0.090	0.059	–	–
Threshold	1167.44	48	0.946	0.084	0.059	−0.002	−0.006
Metric	955.53	55	0.957	0.070	0.059	0.011	−0.014
Scalar	1039.36	62	0.953	0.069	0.060	−0.004	−0.001
**Age groups (** ***n*** ** = 6690)**							
Configural	1076.32	80	0.953	0.086	0.059	–	–
Threshold	1144.42	104	0.951	0.077	0.059	−0.002	−0.009
Metric	991.06	125	0.959	0.064	0.060	0.008	−0.013
Scalar	1327.02	146	0.944	0.070	0.061	−0.015	0.005
**Educational level (** ***n*** ** = 6652)**							
Configural	1068.75	60	0.951	0.087	0.058	–	–
Threshold	1151.73	76	0.947	0.080	0.058	−0.003	−0.007
Metric	915.90	90	0.960	0.064	0.059	0.012	−0.016
Scalar	1041.78	104	0.954	0.064	0.059	−0.005	−0.001
**P3CEQ-CC subscale**							
**Gender (** ***n*** ** = 6680)**							
Configural	262.76	10	0.951	0.087	0.057	–	–
Threshold	288.75	13	0.947	0.080	0.057	−0.004	−0.007
Metric	244.85	17	0.956	0.063	0.057	0.009	−0.016
Scalar	255.50	19	0.955	0.061	0.057	−0.002	−0.002
**Age groups (** ***n*** ** = 6680)**							
Configural	269.99	20	0.953	0.087	0.058	–	–
Threshold	308.37	29	0.947	0.076	0.058	−0.006	−0.011
Metric	265.02	41	0.958	0.057	0.059	0.010	−0.019
Scalar	441.82	47	0.925	0.071	0.060	−0.032	0.014
**Educational level (** ***n*** ** = 6642)**							
Configural	260.25	15	0.953	0.086	0.057	–	–
Threshold	305.77	21	0.946	0.078	0.057	−0.008	−0.008
Metric	249.25	29	0.958	0.059	0.057	0.012	−0.020
Scalar	454.90	33	0.920	0.076	0.063	−0.038	0.017
**PNS Factor 1: Active engagement**							
**Gender (** ***n*** ** = 6680)**							
Configural	140.63	4	0.993	0.101	0.025	–	–
Threshold	116.67	12	0.994	0.051	0.025	0.002	−0.050
Metric	100.69	15	0.995	0.041	0.025	0.001	−0.010
Scalar	106.19	18	0.995	0.038	0.025	0.000	−0.003
**Age groups (** ***n*** ** = 6680)**							
Configural	166.06	8	0.992	0.109	0.025	–	–
Threshold	156.78	32	0.993	0.048	0.025	0.002	−0.060
Metric	143.61	41	0.995	0.039	0.026	0.001	−0.010
Scalar	173.74	50	0.993	0.039	0.025	−0.001	0.000
**Educational level (** ***n*** ** = 6642)**							
Configural	150.07	6	0.991	0.104	0.026	–	–
Threshold	167.69	22	0.991	0.055	0.026	0.000	−0.049
Metric	154.49	28	0.992	0.045	0.027	0.001	−0.010
Scalar	165.65	34	0.992	0.042	0.027	0.000	−0.003
**PNS Factor 3: Reliance and cooperation with health professionals**							
**Gender (** ***n*** ** = 6683)**							
Configural	229.88	4	0.960	0.130	0.041	–	–
Threshold	265.57	12	0.955	0.080	0.041	−0.005	−0.050
Metric	225.36	15	0.963	0.065	0.042	0.008	−0.015
Scalar	465.14	18	0.921	0.086	0.043	−0.042	0.021
**Age groups (** ***n*** ** = 6683)**							
Configural	243.49	8	0.959	0.133	0.043	–	–
Threshold	305.47	32	0.952	0.072	0.043	−0.007	−0.061
Metric	272.07	41	0.960	0.058	0.045	0.007	−0.013
Scalar	655.10	50	0.895	0.085	0.045	−0.065	0.027
**Educational level (** ***n*** ** = 6645)**							
Configural	222.95	6	0.962	0.128	0.041	–	–
Threshold	280.03	22	0.955	0.073	0.041	−0.007	−0.055
Metric	270.43	28	0.957	0.063	0.044	0.003	−0.010
Scalar	730.26	34	0.878	0.096	0.044	−0.080	0.034
**MCBS + eHEALS**							
**Gender (** ***n*** ** = 6680)**							
Configural	1067.22	10	0.959	0.178	0.069	–	–
Threshold	1081.94	15	0.959	0.146	0.069	0.000	−0.032
Metric	849.80	19	0.968	0.114	0.069	0.009	−0.032
Scalar	810.59	23	0.970	0.101	0.069	0.002	−0.013
**Age groups (** ***n*** ** = 6680)**							
Configural	1093.97	20	0.959	0.179	0.069	–	–
Threshold	1113.08	35	0.959	0.136	0.069	0.000	−0.044
Metric	866.52	47	0.969	0.102	0.070	0.010	−0.034
Scalar	880.43	59	0.969	0.091	0.070	0.000	−0.011
**Educational level (** ***n*** ** = 6642)**							
Configural	1078.10	15	0.958	0.179	0.070	–	–
Threshold	1102.40	25	0.957	0.140	0.070	−0.001	−0.039
Metric	858.60	33	0.967	0.106	0.071	0.010	−0.033
Scalar	922.09	41	0.965	0.099	0.072	−0.002	−0.008
**MCBS**							
**Gender (** ***n*** ** = 6678)**							
Configural	835.08	4	0.967	0.249	0.067	–	–
Threshold	842.62	8	0.966	0.177	0.067	0.000	−0.073
Metric	707.94	11	0.972	0.138	0.067	0.006	−0.039
Scalar	678.49	14	0.973	0.119	0.067	0.001	−0.019
**Age groups (** ***n*** ** = 6678)**							
Configural	871.48	8	0.965	0.254	0.067	–	–
Threshold	873.88	20	0.966	0.160	0.067	0.000	−0.094
Metric	735.98	29	0.972	0.121	0.068	0.006	−0.039
Scalar	747.05	38	0.972	0.106	0.068	0.000	−0.015
**Educational level (** ***n*** ** = 6640)**							
Configural	822.19	6	0.966	0.248	0.067	–	–
Threshold	837.83	14	0.966	0.163	0.067	0.000	−0.085
Metric	709.48	20	0.971	0.125	0.067	0.006	−0.038
Scalar	755.34	26	0.970	0.113	0.067	−0.002	−0.012

*Abbreviations*: *χ²* chi-square statistic, *df* degrees of freedom, *CFI* Comparative Fit Index, *RMSEA* Root Mean Square Error of Approximation, *SRMR* Standardised Root Mean Square Residual, *PROMIS GPH* Patient-Reported Outcomes Measurement Information System Global Physical Health, *PROMIS GMH* Patient-Reported Outcomes Measurement Information System Global Mental Health, *WHO-5* WHO-5 Well-Being Index, *P3CEQ* Person-Centred Coordinated Care Experience Questionnaire, *P3CEQ-PC* P3CEQ Person-Centredness subscale, *P3CEQ-CC* P3CEQ Care Coordination subscale, *PNS* Porter–Novelli Scale, *MCBS* Medicare Current Beneficiary Survey, *eHEALS* eHealth Literacy Scale, *WLSMV* weighted least squares with mean and variance adjustment

Models were estimated using WLSMV with indicators treated as ordered categorical

ΔCFI and ΔRMSEA were calculated as the fit index for the more constrained model minus that for the preceding model. Invariance was supported when ΔCFI ≥ −0.010 and ΔRMSEA ≤ 0.015

For the WHO-5 age-group analysis, the two lowest response categories of item 2 were collapsed because the lowest category was unobserved in one age group

Scalar invariance was supported for PROMIS GPH across gender and educational level, but not age group. For PROMIS GMH, scalar invariance was supported across gender and educational level, but not age group. WHO-5 met scalar criteria across all three grouping variables.

Scalar invariance was supported for the P3CEQ total scale across gender but not age group or educational level. For P3CEQ-PC, scalar criteria were met across gender and educational level but not age group, whereas P3CEQ-CC met scalar criteria across gender only.

PNS Factor 1 met scalar criteria across all grouping variables, whereas PNS Factor 3 did not meet scalar criteria across any grouping variable. PNS Factor 2 was not tested because its two-item structure was considered insufficient for conventional multigroup CFA.

Both self-management confidence specifications met scalar criteria across all three grouping variables.

### Item response theory analyses

IRT results are shown in Table 7. An overview of the test information curves is provided in Supplementary Figure S1, with model-specific IRT curves presented in Supplementary Figures S2–S11. The test information curves showed differences in the magnitude and distribution of measurement information across the models, with maximum precision occurring at different regions of the respective latent traits. Item-level S-X² tests indicated statistically significant misfit for most items across models, including items within the otherwise stronger PROM models (Table 7).

**Table 7:** Graded response model parameter estimates for scales included in the item response theory analyses

	a	b1	b2	b3	b4	b5	S-X²	*p*
**PROMIS GPH (** ***n*** ** = 6588)**								
PROMIS GPH 1 Physical health	2.508	-1.548	-0.358	1.206	2.541		41.234	0.001
PROMIS GPH 2 Everyday physical functioning	2.544	-2.817	-1.745	-1.025	-0.257		58.465	<0.001
PROMIS GPH 3 Average fatigue	2.069	-3.594	-1.926	-0.288	0.838		70.279	<0.001
PROMIS GPH 4 Average pain	1.584	-4.431	-1.667	-0.141	1.579		69.571	<0.001
**PROMIS GMH (** ***n*** ** = 6603)**								
PROMIS GMH 1 Quality of life	2.277	-2.070	-0.886	0.544	2.078		77.591	<0.001
PROMIS GMH 2 Mental health	3.291	-2.108	-1.098	0.058	1.266		185.302	<0.001
PROMIS GMH 3 Satisfaction with social activities and relationships	2.595	-2.013	-1.048	0.278	1.637		59.923	<0.001
PROMIS GMH 4 Emotional problems	1.828	-3.506	-1.626	-0.165	1.298		117.878	<0.001
**WHO-5 (** ***n*** ** = 6598)**								
WHO-5 1 Feeling cheerful and in good spirits	3.685	-2.751	-1.592	-0.966	-0.177	1.575	132.242	<0.001
WHO-5 2 Feeling calm and relaxed	3.127	-2.705	-1.679	-0.960	-0.181	1.480	315.969	<0.001
WHO-5 3 Feeling active and vigorous	2.480	-1.987	-1.175	-0.437	0.316	1.854	171.497	<0.001
WHO-5 4 Interest in daily life	2.522	-3.080	-1.587	-0.908	-0.003	1.713	155.752	<0.001
WHO-5 5 Feeling refreshed on waking	2.084	-1.949	-1.157	-0.431	0.268	1.917	106.710	<0.001
**P3CEQ total scale (** ***n*** ** = 5009)**								
P3CEQ 1 Discussion of what matters most	1.383	-3.201	-1.251	0.586			145.974	<0.001
P3CEQ 2 Involvement in care decisions	2.053	-2.911	-1.852	-0.190			78.637	0.001
P3CEQ 3 Being treated as a whole person	2.316	-2.609	-1.730	-0.528			59.040	0.042
P3CEQ 4 Having to repeat information	0.986	-3.002	-1.506	0.682			105.068	<0.001
P3CEQ 5 Care organised to meet patient needs	2.524	-2.557	-1.695	-0.239			64.025	0.009
P3CEQ 6 Single professional coordinating care	0.781	0.212	NA	NA			38.120	0.009
P3CEQ 7 Care plan addressing patient needs	0.819	1.101	NA	NA			29.401	0.044
P3CEQ 8 Support for self-management	1.179	-1.399	-0.482	0.533			346.183	<0.001
P3CEQ 9 Timely and useful self-management information	1.049	-1.095	-0.139	1.434			611.625	<0.001
P3CEQ 10 Confidence in managing own health	0.727	-3.675	0.208	2.965			90.525	0.001
**P3CEQ-PC subscale (** ***n*** ** = 5053)**								
P3CEQ 1 Discussion of what matters most	1.296	-3.347	-1.308	0.598			122.316	<0.001
P3CEQ 2 Involvement in care decisions	2.141	-2.855	-1.825	-0.196			56.439	0.044
P3CEQ 3 Being treated as a whole person	2.440	-2.555	-1.702	-0.528			44.762	0.209
P3CEQ 4 Having to repeat information	1.064	-2.834	-1.428	0.645			106.230	<0.001
P3CEQ 5 Care organised to meet patient needs	2.502	-2.567	-1.708	-0.252			66.353	0.003
P3CEQ 8 Support for self-management	1.186	-1.401	-0.495	0.521			356.267	<0.001
P3CEQ 9 Timely and useful self-management information	0.960	-1.169	-0.153	1.520			706.029	<0.001
P3CEQ 10 Confidence in managing own health	0.784	-3.446	0.194	2.785			58.982	0.079
**P3CEQ-CC subscale (** ***n*** ** = 6088)**								
P3CEQ 5 Care organised to meet patient needs	1.608	-3.140	-2.049	-0.282			190.030	<0.001
P3CEQ 6 Single professional coordinating care	1.039	0.172					99.587	<0.001
P3CEQ 7 Care plan addressing patient needs	1.199	0.842					32.096	<0.001
P3CEQ 8 Support for self-management	1.253	-1.310	-0.411	0.542			542.344	<0.001
P3CEQ 9 Timely and useful self-management information	1.350	-0.904	-0.081	1.239			394.568	<0.001
**PNS Factor 1: Active engagement (** ***n*** ** = 6599)**								
PNS 3 Active disease prevention	1.329	-3.770	-3.061	-1.169	1.364		255.068	<.001
PNS 5 Importance of health information	2.505	-2.666	-2.425	-1.304	0.509		179.916	<.001
PNS 6 Health information for oneself and family	3.024	-2.613	-2.272	-1.142	0.595		72.793	<.001
PNS 10 Understanding personal health risks	2.007	-3.229	-2.883	-1.767	0.898		145.488	<.001
**PNS Factor 3: Reliance and cooperation with health professionals (** ***n*** ** = 6587)**								
PNS 1 Reliance on professionals for health information	1.153	-3.036	-2.028	-0.557	1.480		297.021	<0.001
PNS 4 Reliance on professionals for health decisions	0.683	-3.037	-0.807	1.401	4.598		276.627	<0.001
PNS 8 Working with professionals to manage health	2.978	-2.221	-1.558	-0.551	1.021		146.486	<0.001
PNS 9 Discussing relevant information with professionals	1.131	-3.414	-1.847	0.176	2.620		193.629	<0.001
**MCBS + eHEALS (** ***n*** ** = 4802)**								
MCBS 1 Confidence in following home-care instructions	3.270	-0.612	0.587	2.151			160.069	<0.001
MCBS 2 Confidence in following lifestyle-change instructions	2.667	-1.117	0.271	1.802			156.682	<0.001
MCBS 3 Confidence in recognising need for medical care	1.787	-1.121	0.390	2.264			81.247	<0.001
MCBS 4 Confidence in recognising medication side effects	1.357	-1.943	-0.252	1.624			79.738	<0.001
eHEALS Confidence in using online health information for decisions	0.779	-4.349	-1.893	0.668			250.835	<0.001
**MCBS (** ***n*** ** = 5920)**								
MCBS 1 Confidence in following home-care instructions	3.513	-0.605	0.578	2.122			192.931	<.001
MCBS 2 Confidence in following lifestyle-change instructions	2.749	-1.110	0.267	1.788			190.924	<.001
MCBS 3 Confidence in recognising need for medical care	1.708	-1.146	0.398	2.321			106.556	<.001
MCBS 4 Confidence in recognising medication side effects	1.283	-2.011	-0.263	1.682			193.320	<.001

*Abbreviations*: *PROMIS GPH* Patient-Reported Outcomes Measurement Information System Global Physical Health, *PROMIS GMH* Patient-Reported Outcomes Measurement Information System Global Mental Health, *WHO-5* WHO-5 Well-Being Index, *P3CEQ* Person-Centred Coordinated Care Experience Questionnaire, *P3CEQ-PC* P3CEQ Person-Centredness subscale, *P3CEQ-CC* P3CEQ Care Coordination subscale, *PNS* Porter–Novelli Scale, *MCBS* Medicare Current Beneficiary Survey, *eHEALS* eHealth Literacy Scale, *NA* not applicable

a = discrimination parameter; b1–b5 = category thresholds; S-X² = item-level fit statistic; *p *< 0.05 indicates lack of fit

PROMIS GPH showed the strongest IRT support, with good global fit and high to very high discrimination. PROMIS GMH and WHO-5 showed mixed but broadly acceptable fit, with elevated RMSEA but good CFI and SRMSR values, ordered thresholds, and very high discrimination. Across the PROMIS and WHO-5 models, information was concentrated at lower to moderate trait levels, with reduced precision at higher levels (Supplementary Figures S1–S4).

The P3CEQ models showed mixed IRT performance. The total scale had suboptimal global fit and variable discrimination, with P3CEQ6, P3CEQ7 and P3CEQ10 contributing least information. P3CEQ-PC provided more information than P3CEQ-CC, while both subscales showed reduced precision at higher trait levels (Supplementary Figures S5–S7).

For the multidimensional PNS, Factors 1 and 3 were modelled separately; PNS Factor 2 was not modelled because it comprised only two items. PNS Factor 1 showed stronger IRT support than PNS Factor 3, with better fit and more consistent discrimination. Within PNS Factor 3, PNS8 contributed substantially more information than the other items (Supplementary Figures S8–S9).

The self-management confidence specifications showed the weakest IRT support. Both the MCBS + eHEALS and MCBS-only models had suboptimal global fit, with high RMSEA and lower CFI values than the PROM models, and variable discrimination. Their information was concentrated primarily around the central to moderately higher regions of the respective latent traits. The eHEALS item showed moderate discrimination and contributed least information in the five-item model, while MCBS4 showed moderate discrimination and contributed least information in the MCBS-only model (Supplementary Figures S10–S11).

Local item dependence was generally limited but was identified for selected item pairs, with the clearest pattern occurring in the self-management confidence models (Supplementary Table S2).

### Construct validity

The pattern of inter-scale correlations was broadly consistent with expectations (Table 8). The PROMs were strongly intercorrelated, whereas PROM–PREM associations were weak to moderate. The P3CEQ total scale and P3CEQ-PC and P3CEQ-CC subscales were highly intercorrelated. Associations involving capability-related measures were generally weaker: PROM–PNS correlations were weak, whereas P3CEQ–PNS correlations were weak to moderate. The two self-management confidence specifications were almost perfectly correlated (*r* = 0.97). Correlations with the reverse-coded self-management confidence scores were negative and weak to moderate for both PROMs and PREM, indicating that higher PROM and PREM scores were associated with greater self-management confidence.

**Table 8:** Pearson correlations between scale scores

	PROMIS GPH	PROMIS GMH	WHO-5	P3CEQ total scale	P3CEQ-PC subscale	P3CEQ-CC subscale	PNS Factor 1	PNS Factor 2	PNS Factor 3	MCBS	MCBS + eHEALS
**PROMIS GPH**	1.000	0.588	0.690	0.196	0.293	0.084	0.133	0.257	-0.035	-0.274	-0.262
**PROMIS GMH**		1.000	0.777	0.260	0.342	0.140	0.135	0.242	0.027	-0.330	-0.320
**WHO-5**			1.000	0.292	0.360	0.185	0.111	0.174	0.072	-0.292	-0.286
**P3CEQ total scale**				1.000	0.920	0.920	0.208	0.216	0.450	-0.402	-0.381
**P3CEQ-PC subscale**					1.000	0.744	0.235	0.294	0.421	-0.477	-0.453
**P3CEQ-CC subscale**						1.000	0.138	0.102	0.423	-0.240	-0.222
**PNS Factor 1**							1.000	0.284	0.252	-0.339	-0.331
**PNS Factor 2**								1.000	-0.049	-0.436	-0.435
**PNS Factor 3**									1.000	-0.190	-0.174
**MCBS**										1.000	0.966
**MCBS + eHEALS**											1.000

*Abbreviations*: *PROMIS GPH* Patient-Reported Outcomes Measurement Information System Global Physical Health, *PROMIS GMH* Patient-Reported Outcomes Measurement Information System Global Mental Health, *WHO-5* WHO-5 Well-Being Index, *P3CEQ* Person-Centred Coordinated Care Experience Questionnaire, *P3CEQ-PC* P3CEQ Person-Centredness subscale, *P3CEQ-CC* P3CEQ Care Coordination subscale, *PNS* Porter–Novelli Scale; MCBS, Medicare Current Beneficiary Survey, *eHEALS* eHealth Literacy Scale, *PROM* patient-reported outcome measure, *PREM* patient-reported experience measure

For the MCBS and MCBS + eHEALS scores, higher values indicate lower self-management confidence; thus, negative correlations with PROM and PREM scores indicate that higher PROM and PREM scores are associated with greater self-management confidence

## Discussion

This study evaluated the psychometric performance of the multi-item scales included in the Norwegian PaRIS-PQ in a large national sample of people with chronic conditions receiving primary care. Overall, psychometric performance varied across scales. The PROMs showed the strongest and most consistent support, whereas the PREM and capability-oriented measures showed more complex measurement properties. The findings support scale-specific interpretation, especially for subgroup comparisons and latent scale use.

The PROMIS physical and mental health scales and the WHO-5 performed best overall. PROMIS GPH showed strong confirmatory support, with CFI, TLI and SRMR meeting the reference criteria and RMSEA slightly exceeding the criterion, while PROMIS GMH and WHO-5 showed strong factor loadings, acceptable internal consistency and broadly supportive, although mixed, CFA results. The elevated RMSEA values, particularly for PROMIS GMH and WHO-5, should therefore be interpreted alongside the stronger CFI, SRMR, factor loading and internal consistency results. Overall, this pattern is consistent with prior evidence supporting the two-summary-score structure of PROMIS Global Health and the unidimensional structure of WHO-5, and supports their use for assessing physical health, mental health and well-being within the PaRIS conceptual framework and PaRIS-10 indicator set [2, 6, 10, 19, 20].

The P3CEQ showed weaker and less stable measurement properties. The total scale showed suboptimal CFA fit, and scalar invariance was only partly supported, with support across gender but not age group or educational level. Subscale results were also uneven, with weaker internal consistency for P3CEQ-CC. This is consistent with the original UK validation, which noted overlap between person-centredness and coordination items and challenges related to response formats [21], and with the PaRIS Field Trial, which pointed more strongly to a dominant general factor than to a clearly separable two-factor structure (9, 10). The Norwegian findings suggest that neither the total scale nor the current subscale specification is fully robust, supporting further refinement of item overlap, response formats and scoring.

The PNS showed a more differentiated and uneven structure. The international Field Trial reported two factors reflecting active engagement and reliance on health professionals [9, 10], whereas the Norwegian EFA supported three factors. PNS Factor 1, reflecting active engagement, showed the strongest psychometric support and stable cross-group measurement properties. PNS Factor 3 performed less well, with lower reliability, no scalar invariance and weaker IRT fit, and its stronger correlation with P3CEQ suggests that it may partly capture patients’ evaluations of their relationship with, or support from, health professionals. PNS Factor 2, reflecting health information comprehension, comprised only two items, which limited further psychometric testing, but the same two PNS-derived items have been operationalised as a health literacy measure in recent Norwegian PaRIS analyses and were associated with PREMs and PROMs [14]. The PNS should therefore be interpreted as multidimensional and partly exploratory rather than as a single robust reflective scale, consistent with the original Porter–Novelli Scale as a brief screening tool and with the Field Trial recommendation for further work on cross-country variation in health information and decision-making preferences [10, 22].

The self-management confidence item sets also showed mixed support. The PaRIS Field Trial evaluated a broader self-management confidence construct comprising P3CEQ10, four MCBS items and one eHEALS-derived item [9, 10], whereas the present study retained P3CEQ10 within the P3CEQ analyses and evaluated the MCBS + eHEALS and MCBS-only specifications separately. Both specifications showed exploratory support, but CFA and IRT provided limited support for robust unidimensional latent measurement. Removing eHEALS did not improve model fit, while both specifications met scalar invariance criteria across gender, age group and educational level. Local item dependence was also most evident for the self-management confidence specifications, particularly between MCBS3 and MCBS4, which may inflate reliability and information estimates, further supporting cautious interpretation of these item sets.

The weaker performance of some experience- and capability-related measures should be interpreted in relation to the reflective specifications evaluated. This is particularly relevant for the PNS and self-management confidence item sets, which were not originally developed as conventional unidimensional scales in their present PaRIS configurations [9, 10, 22]. Weaker fit therefore indicates limited support for the reflective model tested rather than poor relevance of the individual items. Conversely, poor reflective-model fit does not by itself establish a formative measurement model, which would require separate conceptual justification and empirical evaluation [45].

Item-level fit provided an additional qualification. S-X² indicated statistically significant misfit for most items, including within the otherwise stronger PROM models. Given the large analytical sample, the chi-square-based S-X² tests may also have detected relatively small departures from the GRM as statistically significant. These findings were therefore interpreted in conjunction with global model fit, discrimination, information and local dependence rather than in isolation. The widespread item-level misfit indicates that further item-level evaluation is warranted across the PaRIS-PQ scales, including measures with otherwise supportive scale-level performance.

Measurement invariance and construct validity have practical implications for PaRIS-PQ scale use. Metric invariance was supported for all tested models, supporting analyses of associations across demographic groups. Scalar invariance was less consistent across measures. PROMIS GPH and PROMIS GMH met scalar criteria across gender and educational level but not age group. WHO-5, PNS Factor 1 and both self-management confidence specifications met scalar criteria across all grouping variables. Scalar support was more variable for the P3CEQ models, whereas PNS Factor 3 did not meet scalar criteria across any grouping variable. Correlations supported construct validity and related but distinct outcome, experience and capability domains, with strong PROM intercorrelations and weaker PROM–PREM, PROM–capability and PREM–capability associations, consistent with the Field Trial [9, 10].

Cross-country comparability requires separate consideration. Differences in healthcare-system organisation are explicitly part of the contextual variation that PaRIS is designed to examine; nested survey design and the associated conceptual framework link patient-reported outcomes and experiences to practice- and system-level characteristics [6, 7]. The use of common measures and harmonised translation and survey procedures is intended to support cross-country comparison [2, 7, 9] but does not in itself establish measurement invariance [13]. In the Field Trial, multigroup CFA of the PROMs supported configural invariance, whereas metric models showed greater misfit and scalar invariance was not supported [9, 10]. The present study, based on data from Norway only, cannot assess cross-country measurement invariance; this requires analyses using data from multiple countries, which is an important avenue for further research for the OECD PaRIS initiative. Cross-country comparisons of scale scores should be interpreted based on the results from the analysis of the PaRIS database at the international level.

Taken together, the Norwegian findings support scale-specific rather than uniform use of the PaRIS-PQ. They support the PaRIS strategy of combining established scales with additional items while showing that measurement properties vary across domains and require context-specific evaluation [9, 10, 13]. For current use, the PROMIS GPH, PROMIS GMH and WHO-5 have the strongest overall support for monitoring and research, whereas the P3CEQ, PNS factors beyond active engagement, and the exploratory self-management confidence item sets warrant more cautious, purpose-specific interpretation. In particular, subgroup mean comparisons should be interpreted cautiously where scalar invariance was not supported, and latent-scale analyses are less well supported where dimensionality or model fit was mixed. The psychometric evaluation also identifies opportunities to refine item selection, response formats and scale construction in the experience and capability domains. Further analyses using cross-country Main Survey data should examine item-level functioning and measurement invariance to determine whether the observed limitations are country-specific or more general and to guide future refinement and use of PaRIS measures. Future PaRIS cycles should assess scale performance alongside representativeness and provider participation, given Norwegian evidence of GP participation-related selection [12].

### Strengths and limitations

This study provides the first comprehensive psychometric evaluation of the multi-item scales in the Norwegian PaRIS-PQ using Main Survey data. It covers scales underpinning key PaRIS indicators and applies complementary methods, including EFA, CFA, internal consistency assessment, measurement invariance testing and IRT analyses. The large national sample strengthened precision and enabled subgroup analyses, while the provider-linked design was closely aligned with the intended PaRIS implementation model. The study also benefited from the Norwegian translation and cognitive testing process, which supported linguistic and conceptual adaptation before field implementation.

Some limitations should also be noted. First, the evaluation was restricted to multi-item scales; important single-item PaRIS indicators, including trust, overall perceived quality and general health, were outside the scope of the analyses. Second, several scales, subscales and factors contained few items, resulting in low degrees of freedom in some CFA models and constraining both CFA and IRT modelling. CFA fit indices, particularly RMSEA in models with few degrees of freedom, should therefore be interpreted alongside factor loadings, reliability, other psychometric evidence, and the complementary IRT findings. Third, cognitive testing in Norway used three separate protocols rather than the full patient questionnaire and therefore could not fully assess overall cognitive burden. Finally, as the study was conducted in one national context, cross-country measurement invariance could not be assessed.

## Conclusions

The Norwegian PaRIS-PQ showed varied psychometric performance across its multi-item scales. PROMIS GPH, PROMIS GMH and WHO-5 demonstrated the strongest and most consistent support and appear suitable for monitoring and research. The P3CEQ, the PNS beyond the active engagement factor, and the self-management confidence item sets showed more mixed evidence and require cautious, scale-specific interpretation, especially in subgroup comparisons where scalar invariance was not supported and when treated as reflective latent measures. The findings support further refinement of experience and capability-related measures with weaker psychometric support, particularly those related to person-centredness, coordination and self-management support. Evidence from other countries will be important for assessing generalisability within the PaRIS initiative.

## Supplementary Information

Supplementary Material 1: Table S1

Supplementary Material 2: Table S2

Supplementary Material 3: Figure S1-11

## Data Availability

Anonymous data are available from the corresponding author on reasonable request. Public Use Files from the PaRIS patient survey, including anonymised data from Norway, are available through the OECD PaRIS Public Use Files website.

## Abbreviations

PROM: Patient-reported outcome measure
PREM: Patient-reported experience measure
OECD: Organisation for Economic Co-operation and Development
PaRIS: Patient-Reported Indicator Surveys
PaRIS-PQ: PaRIS Patient Questionnaire
PaRIS-PCPQ: PaRIS Primary Care Practice Questionnaire
PaRIS-10: PaRIS ten key indicators
GP: General practitioner
KUHR: Norwegian Control and Payment of Health Reimbursements Database
EMPRO: Evaluating the Measurement of Patient-Reported Outcomes
TRAPD: Translation, Review, Adjudication, Pretesting and Documentation
PROMIS: Patient-Reported Outcomes Measurement Information System
PROMIS GPH: Patient-Reported Outcomes Measurement Information System Global Physical Health
PROMIS GMH: Patient-Reported Outcomes Measurement Information System Global Mental Health
WHO-5: WHO-5 Well-Being Index
P3CEQ: Person-Centred Coordinated Care Experience Questionnaire
PNS: Porter–Novelli Scale
MCBS: Medicare Current Beneficiary Survey
eHEALS: eHealth Literacy Scale
CFA: Confirmatory factor analysis
EFA: Exploratory factor analysis
P3CEQ-PC: P3CEQ Person-Centredness subscale
P3CEQ-CC: P3CEQ Care Coordination subscale
KMO: Kaiser–Meyer–Olkin
WLSMV: Weighted least squares with mean and variance adjustment
CFI: Comparative Fit Index
TLI: Tucker–Lewis Index
RMSEA: Root Mean Square Error of Approximation
SRMR: Standardised Root Mean Square Residual
IRT: Item response theory
GRM: Graded response model
SRMSR: Standardised Root Mean Square Residual
DPIA: Data Protection Impact Assessment
