# Supplementary material for: Psychometric performance of the OECD Patient-Reported Indicator Surveys Patient Questionnaire (PaRIS-PQ) among adults with chronic conditions in Norway
Source: Qual Life Res. 2026 Nov 7;35(12):326. doi: 10.1007/s11136-026-04429-z (PMC13744511; doi:10.1007/s11136-026-04429-z)
Supplement: Supplementary file 1 [file 11136_2026_4429_MOESM1_ESM.docx]

**Supplementary Table S1**: Overview of the multi-item measures evaluated in the Norwegian PaRIS-PQ.

| **Scale** | **Reference** | **No. of items** | **No. of response categories** | **Construct assessed** | **PaRIS domain/type** |
| --- | --- | --- | --- | --- | --- |
| PROMIS GPH | 19 | 4 | 5* | Self-reported physical health and functioning, including overall physical health, physical functioning, fatigue and pain | PROM |
| PROMIS GMH | 19 | 4 | 5 | Self-reported quality of life and mental health, including mood and ability to think, satisfaction with social activities and relationships, and emotional problems | PROM |
| WHO-5 | 20 | 5 | 6† | Mental well-being during the previous two weeks | PROM |
| P3CEQ | 21 | 10 (PC: 8; CC: 5) | 4 for eight items; 2 for two items‡ | Experiences of person-centred and coordinated care | PREM |
| PNS | 22 | 10 | 5 | Preferences and orientations regarding health information and engagement in managing health and health care | Health and healthcare capabilities |
| MCBS + eHEALS | 9, 10, 23, 24 | 5 (4 MCBS + 1 eHEALS) | 4§ | Confidence in selected capabilities for managing health and health care, including use of online health information | Health and healthcare capabilities |

* The PROMIS pain item is administered on a 0–10 scale and recoded to five categories for scoring (0 = 1; 1–3 = 2; 4–6 = 3; 7–9 = 4; 10 = 5).

† WHO-5 has six response categories, scored from 0 to 5.

‡ In the present analyses, eight P3CEQ items had four scored response categories and two items (P3CEQ6 and P3CEQ7) were dichotomous; the P3CEQ-PC and P3CEQ-CC subscales contain overlapping items.

§ The four confidence response categories were used in the analyses; “Does not apply”, available for the medication side-effects item and the eHEALS-derived item, was not included as a scored response category.

**Abbreviations:** PaRIS-PQ, Patient-Reported Indicator Surveys Patient Questionnaire; PROM, patient-reported outcome measure; PREM, patient-reported experience measure; PROMIS, Patient-Reported Outcomes Measurement Information System; GPH, Global Physical Health; GMH, Global Mental Health; WHO-5, WHO-5 Well-Being Index; P3CEQ, Person-Centred Coordinated Care Experience Questionnaire; PC, Person-Centredness; CC, Care Coordination; PNS, Porter–Novelli Scale; MCBS, Medicare Current Beneficiary Survey; eHEALS, eHealth Literacy Scale.
