# Supplementary material for: Psychometric performance of the OECD Patient-Reported Indicator Surveys Patient Questionnaire (PaRIS-PQ) among adults with chronic conditions in Norway
Source: Qual Life Res. 2026 Nov 7;35(12):326. doi: 10.1007/s11136-026-04429-z (PMC13744511; doi:10.1007/s11136-026-04429-z)
Supplement: Supplementary file 2 [file 11136_2026_4429_MOESM2_ESM.docx]

**Supplementary Table S2:** Local item dependence in the graded response models.

| **Scale** | **Items** | **Item pairs, n** | **Mean raw Q3*** | **Maximum adjusted Q3*** | **Flagged pairs, n** | **Flagged item pairs (adjusted Q3)** |
| --- | --- | --- | --- | --- | --- | --- |
| PROMIS GPH | 4 | 6 | −0.255 | 0.105 | 0 | None |
| PROMIS GMH | 4 | 6 | −0.272 | 0.160 | 0 | None |
| WHO-5 | 5 | 10 | −0.209 | 0.240 | 1 | WHO-5 Item 3 and WHO-5 Item 5 (0.240) |
| P3CEQ total scale | 10 | 45 | −0.081 | 0.234 | 1 | P3CEQ6 and P3CEQ7 (0.234) |
| P3CEQ-PC | 8 | 28 | −0.109 | 0.200 | 1 | P3CEQ1 and P3CEQ9 (0.200) |
| P3CEQ-CC | 5 | 10 | −0.156 | 0.166 | 0 | None |
| PNS Factor 1 | 4 | 6 | −0.223 | 0.190 | 0 | None |
| PNS Factor 3 | 4 | 6 | −0.188 | 0.250 | 1 | PNS4 and PNS1 (0.250) |
| MCBS + eHEALS | 5 | 10 | −0.183 | 0.323 | 3 | MCBS Item 3 and MCBS Item 4 (0.323); eHEALS Item and MCBS Item 4 (0.292); eHEALS Item and MCBS Item 3 (0.219) |
| MCBS | 4 | 6 | −0.264 | 0.440 | 1 | MCBS Item 3 and MCBS Item 4 (0.440) |

*Q3 denotes Yen’s residual correlation. Adjusted Q3 values were calculated by subtracting the mean off-diagonal Q3 from each item-pair residual correlation. Adjusted Q3 values above 0.20, based on unrounded estimates, were used as a heuristic screening value for possible local item dependence and not as an absolute statistical criterion.

**Abbreviations**: PROMIS GPH, Patient-Reported Outcomes Measurement Information System Global Physical Health; PROMIS GMH, Patient-Reported Outcomes Measurement Information System Global Mental Health; WHO-5, WHO-5 Well-Being Index; P3CEQ, Person-Centred Coordinated Care Experience Questionnaire; P3CEQ-PC, P3CEQ Person-Centredness subscale; P3CEQ-CC, P3CEQ Care Coordination subscale; PNS, Porter–Novelli Scale; MCBS, Medicare Current Beneficiary Survey; eHEALS, eHealth Literacy Scale.
