# Supplementary material for: Psychometric performance of the OECD Patient-Reported Indicator Surveys Patient Questionnaire (PaRIS-PQ) among adults with chronic conditions in Norway
Source: Qual Life Res. 2026 Nov 7;35(12):326. doi: 10.1007/s11136-026-04429-z (PMC13744511; doi:10.1007/s11136-026-04429-z)
Supplement: Supplementary file 3 [file 11136_2026_4429_MOESM3_ESM.docx]

**Supplementary Figure S1.** Test information curves for all graded response models evaluated in the study, shown using identical axis ranges to facilitate comparison.

**Abbreviations:** PROMIS GPH, Patient-Reported Outcomes Measurement Information System Global Physical Health; PROMIS GMH, Patient-Reported Outcomes Measurement Information System Global Mental Health; WHO-5, WHO-5 Well-Being Index; P3CEQ, Person-Centred Coordinated Care Experience Questionnaire; P3CEQ-PC, P3CEQ Person-Centredness subscale; P3CEQ-CC, P3CEQ Care Coordination subscale; PNS, Porter–Novelli Scale; MCBS, Medicare Current Beneficiary Survey; eHEALS, eHealth Literacy Scale.

**Supplementary Figure S2.** Item response theory curves for the Patient-Reported Outcomes Measurement Information System Global Physical Health (PROMIS GPH).

**Supplementary Figure S3.** Item response theory curves for the Patient-Reported Outcomes Measurement Information System Global Mental Health (PROMIS GMH).

**Supplementary Figure S4.** Item response theory curves for the WHO-5 Well-Being Index (WHO-5).

**Supplementary Figure S5.** Item response theory curves for the Person-Centred Coordinated Care Experience Questionnaire (P3CEQ) total scale.

**Supplementary Figure S6.** Item response theory curves for the P3CEQ Person-Centredness subscale.

**Supplementary Figure S7.** Item response theory curves for the P3CEQ Care Coordination subscale.

**Supplementary Figure S8.** Item response theory curves for the Porter–Novelli Scale (PNS) Factor 1.

**Supplementary Figure S9.** Item response theory curves for the Porter–Novelli Scale (PNS) Factor 3.

**Supplementary Figure S10.** Item response theory curves for the Medicare Current Beneficiary Survey (MCBS) + eHealth Literacy Scale (eHEALS) self-management confidence specification.

**Supplementary Figure S11.** Item response theory curves for the MCBS-only self-management confidence specification.
